# The field evaluation of a push-pull system to control malaria vectors in Northern Belize, Central America

**DOI:** 10.1186/s12936-015-0692-5

**Published:** 2015-04-29

**Authors:** Joseph M Wagman, John P Grieco, Kim Bautista, Jorge Polanco, Ireneo Briceño, Russell King, Nicole L Achee

**Affiliations:** Department of Preventive Medicine and Biometrics, Uniformed Services University of the Health Sciences, 4301 Jones Bridge Road, Bethesda, MD 20814 USA; College of Biological Sciences, Eck Institute for Global Health, University of Notre Dame, Galvin Life Sciences Center, Notre Dame, IN 46556 USA; Ministry of Health, East Block Independence Plaza, Belmopan, Belize

**Keywords:** *Anopheles vestitipennis*, *An. albimanus*, Spatial repellent, Push-pull, Belize, Transfluthrin, CDC light trap

## Abstract

**Background:**

Campaigns for the continued reduction and eventual elimination of malaria may benefit from new and innovative vector control tools. One novel approach being considered uses a push-pull strategy, whereby spatial repellents are used in combination with outdoor baited traps. The desired effect is the behavioural manipulation of mosquito populations to elicit movement of vectors away from people and into traps.

**Methods:**

Here, a prototype push-pull intervention was evaluated using an experimental hut methodology to test proof-of-principle for the strategy against two natural vector populations, *Anopheles albimanus* and *Anopheles vestitipennis,* in Belize, Central America. A Latin square study design was used to compare mosquito entry into experimental huts and outdoor traps across four different experimental conditions: 1) control, with no interventions; 2) pull, utilizing only outdoor traps; 3) push, utilizing only an indoor spatial repellent; and 4) push-pull, utilizing both interventions simultaneously.

**Results:**

For *An. vestitipennis*, the combined use of an indoor repellent and outdoor baited traps reduced average nightly mosquito hut entry by 39% (95% CI: [0.37 – 0.41]) as compared to control and simultaneously increased the nightly average densities of *An. vestitipennis* captured in outdoor baited traps by 48% (95% CI: [0.22 – 0.74]), compared to when no repellent was used. Against *An. albimanus*, the combined push-pull treatment similarly reduced hut entry, by 54% (95% CI: [0.40 – 0.68]) as compared to control; however, the presence of a repellent indoors did not affect overall outdoor trap catch densities for this species. Against both anopheline species, the combined intervention did not further reduce mosquito hut entry compared to the use of repellent alone.

**Conclusions:**

The prototype intervention evaluated here clearly demonstrated that push-pull strategies have potential to reduce human-vector interactions inside homes by reducing mosquito entry, and highlighted the possibility for the strategy to simultaneously decrease human-vector interactions outside of homes by increasing baited trap collections. However, the variation in effect on different vectors demonstrates the need to characterize the underlying behavioral ecology of target mosquitoes in order to drive local optimization of the intervention.

**Electronic supplementary material:**

The online version of this article (doi:10.1186/s12936-015-0692-5) contains supplementary material, which is available to authorized users.

## Background

Recent achievements in decreasing the global burden of human malaria have come about through the implementation of well-coordinated, multi-faceted and evidence-based control programs of which vector control has been an integral component [1-7]. Indeed, vector control is widely recognized as an essential part of any viable plan to further control, eliminate and eradicate malaria [1,7,8]. However, current adult vector control tools, such as indoor residual spraying (IRS) and long-lasting insecticidal nets (LLINs), are becoming increasingly inadequate to control disease for a variety of reasons, among which are the emergence of insecticide resistance, vector behaviors (e.g. daytime or outdoor-biting) that result in reduced intervention efficacy, and local shifts in vector species composition [1,9-13]. These inadequacies, coupled with renewed calls for the global elimination and eradication of malaria in all of its complex transmission settings, underscore the critical need for novel approaches for vector control [6,14,15].

One novel strategy currently being developed utilizes a push-pull approach, which seeks to exploit the complementary effects of spatial repellents and mosquito traps, used in combination, to decrease the probability of human-vector interactions [16-19]. Developed initially as a way to control agricultural and urban pests, push-pull interventions work by combining the repellency action of one component and the attractiveness of another in order to elicit the movement of pests away from a protected resource and towards a trap for subsequent removal from the environment [18,20,21]. Accordingly, push-pull strategies for the control of mosquito vectors of human disease would use repellents to deter host-seeking mosquitoes from treated spaces (the ‘push’) and towards a baited trap (the ‘pull’), which would result in their capture and removal from the peridomestic environment and thereby decrease population densities for added protection in the outdoor environment [16-18].

Although still in the proof-of-concept phase, it is easy to appreciate that the dynamics of such a strategy are complex and likely to vary according to local transmission ecologies. Nonetheless, preliminary work has been encouraging. For example, Kitau *et al.* showed in a semi-field environment that the combined use of personal repellents (topically applied) and mosquito traps could reduce the biting rates of laboratory reared *Anopheles gambiae* more than the use of traps alone [17] and Menger *et al.*, also working with *An. gambiae* in a semi-field setup, recently showed that a combination of spatial repellents and baited traps can be used to reduce mosquito house entry [19]. Additionally, a number of researchers [22-24] have made progress towards defining the parameters of a push-pull intervention for the control of the dengue vector *Aedes aegypti*, including studies demonstrating local interest in and community acceptance of the concept in both Latin America and South East Asia [16]. In order to assess the potential role for push-pull strategies in the prevention of malaria, the present pilot study measured the impact of a prototype push-pull intervention on natural populations of two regionally important malaria vectors in Belize, Central America: *Anopheles albimanus* and *Anopheles vestitipennis* [25,26]. A field based, matched-control experimental hut study design was used to measure and compare two important endpoints, 1) the reduction of host-seeking mosquito entry into the huts and 2) the numbers of mosquitoes collected in the outdoor baited light traps.

## Methods

### Ethics statement

Permits and approval for this study were obtained from the Ministry of Health, Belize (IRB 01/12(02)) and the Pesticides Control Board, Belize (Ref. PCB/EXP/MOH/01/12). No protected species were sampled during these studies.

### Study site and design

**Figure 1:**
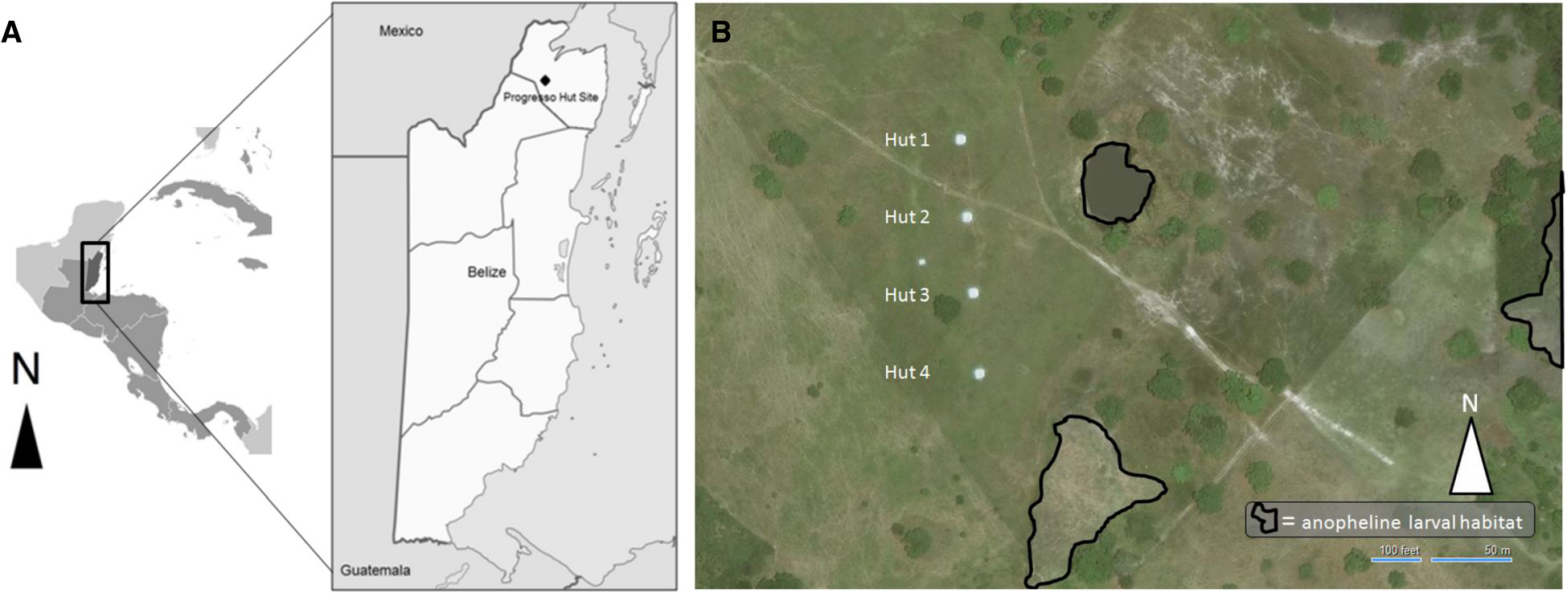
The experimental field site. **(A)** The location of Belize in Central America, and the location of the site near Progresso Village **(B)** Satellite imagery of the site showing the experimental huts locations and with the nearest anopheline larval habitats indicated.

The study site was established in an open pasture surrounded by freshwater lagoons and seasonal swampland near the village of Progresso in Corozal District, Belize (N18°11’52“ W88°26’6”) (Figure 1A). A Latin square study design was used to compare mosquito entry into experimental huts and outdoor traps across four different experimental conditions: 1) control, with no interventions; 2) ‘pull,’ utilizing only outdoor traps; 3) ‘push,’ utilizing only an indoor spatial repellent; and 4) ‘push-pull,’ utilizing both interventions simultaneously. Experimental treatments and collection teams were independently rotated through each of the four huts with each specific combination occurring exactly once, a total replication of 16 nights (Additional file 1). Collections were carried out on non-consecutive nights during the rainy season of 2012, which corresponds to the annual period of peak anopheline densities in the region [27-29]. Baseline collections lasted from July to August and experimental collections from September to November, during which the region experienced an average of 160 mm/month of precipitation (range 305 mm/month in August to 50 mm/month in December) [30]. Temperature and humidity inside the huts were measured using HOBO® Pro Series Weatherproof Data Loggers (Forestry Suppliers Inc., Jackson, MS). Wind speed, relative humidity, temperature, and precipitation were recorded outdoors with a Davis Vantage Vue® wireless weather station (Davis Instruments, Vernon Hills, IL).

### Experimental huts and interception traps

**Figure 2:**
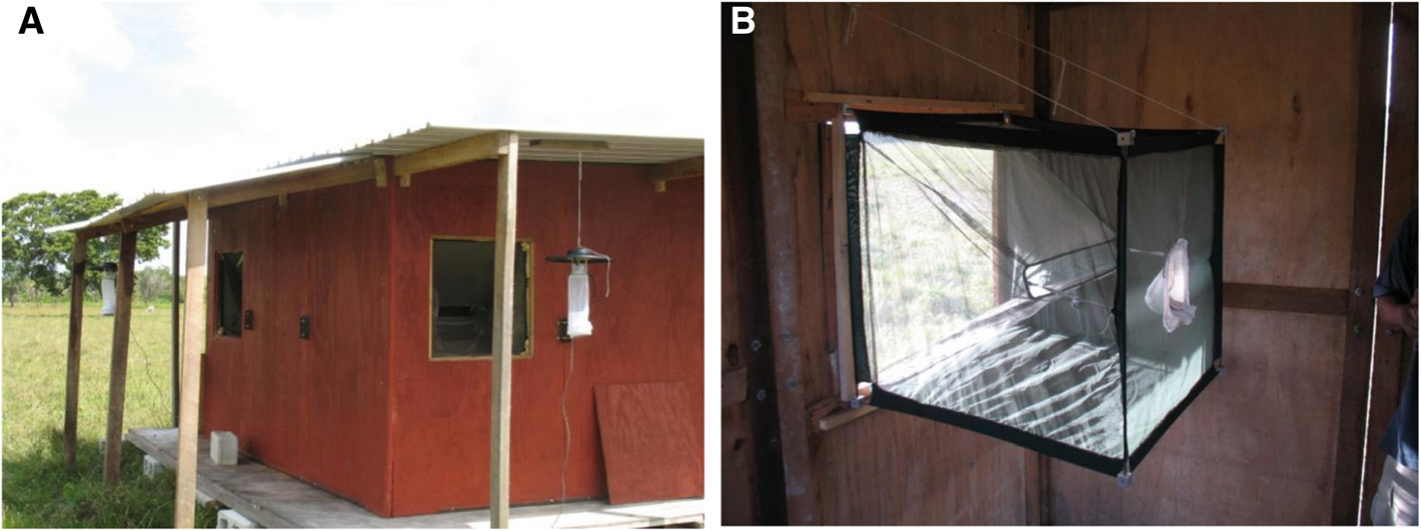
Hut configuration. **(A)** An experimental hut at the field site and **(B)** a window interception trap inside the same hut.

Four identical experimental huts were constructed on site, approximately 50 meters apart along a straight, north-south transect (Figure 1B). Based on previously described methodologies [31], huts were built using locally acquired materials and in a style typical of homes in rural Belize (Figure 2A). Briefly, each structure measured 3.6 m × 3.6 m and had an average roof height of 2.36 m, creating an internal volume of roughly 30.6 m^3^. Huts were raised 30 cm from the ground, resting on a cinderblock and pine wood platform. Walls and floors were constructed of an untreated pine lumber frame with plywood panels. Roofs were fashioned out of corrugated, galvanized steel panels. Each hut had one door (182 cm × 76.2 cm), cut into the eastern facing wall, and three windows (76.2 cm × 76.2 cm), one in each of the remaining walls. Windows were built to accommodate interception traps for capturing mosquitoes entering the hut (Figure 2B). Based on the designs of Muirhead-Thomson and by Grieco *et al.* [32,33], interception traps measured 76.2 cm × 76.2 cm × 76.2 cm and were made of a steel frame (3.2 mm diameter rebar) covered with a green polyester netting (BioQuip Products Inc., Rancho Dominguez, CA) bag. A beveled opening prevented trapped mosquitoes from escaping, while a 20 cm diameter portal enabled the aspiration of trapped specimens from inside the hut. White polyethylene tarpaulin sheets (A&R Enterprises, LTD, Belize City, BZ) were installed on hut floors and baseboards to facilitate the monitoring of knocked down mosquitoes. To control for residual chemical contamination from repellent treatments, all huts and interception traps were cleaned after every four collections, prior to the rotation of treatments among huts. Hut surfaces and trap netting were sprayed and washed with a 10% bleach solution and windows and doors were left open for 24 hours.

### Outdoor baited traps

**Figure 3:**
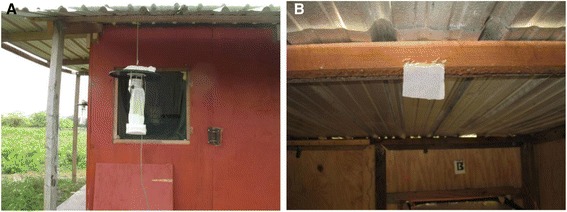
Experimental treatments. **(A)** An outdoor baited CDC light trap and **(B)** a spatial repellent emanator inside an experimental hut.

Preliminary (2011) trials at the field site indicated that CDC Miniature Light Traps (CDC LT) (John W. Hock Company, Gainesville, FL), baited with human foot emanations collected on cotton socks [34,35], captured the greatest numbers of *An. vestitipennis* and *An. albimanus* [29]. Prior to use as a mosquito lure all socks were worn for 12 h by the same individual and were utilized for a maximum of 72 h after initial collection. During use, socks were placed on top of the CDC LT rain guard, and when not in use, were stored away from sunlight in sealed plastic bags at ambient temperature. During the collections, baited CDC LTs were positioned and operated according to manufacturer’s recommendations and previous methodologies. Traps were hung outside the huts, 2 m above the ground and 1 m from each of the three open windows (Figure 3A) [29]. Traps were baited, positioned and turned on 30 min before sunset (~1730 h) and operated until shortly after sunrise (~0600 h). During each 12 h replicate, CDC trap bags were replaced with clean bags every 2 h by the on-site study coordinator, who was working from a central processing station located approximately 60 m from the experimental hut transect. Collection bag contents were sorted and captured mosquitoes were stored in plastic collection cups labeled by time, hut and unique CDC LT identifier.

### Spatial repellent

Transfluthrin (S.C. Johnson and Son, Inc., Racine WI) (2,3,5,6-tetrafluorobenzyl (1R)-trans-3-(2,2-dichlorovinyl)-2,2-dimethylcyclopropanecarboxylate), a volatile synthetic pyrethroid with spatial repellent (SR) efficacy against several classes of arthropod pests including anopheline mosquitoes [36-38], was selected as the chemical repellent. Following industry guidelines for a recommended dosage of 30 mg active ingredient per 9.3 m^2^ area (M.C. Meier, personal communication, 16 August 2011) each experimental hut (13.4 m^2^ floor space) received a total 43.2 mg of transfluthrin emanating passively from two 55.6 cm^2^ strips of nylon organdy cloth (G-Street Fabrics, Bethesda MD). Each cloth strip was treated with 21.6 mg of technical grade transfluthrin diluted in 1 mL of acetone (Ace Hardware Corp., Oak Brook, Illinois) applied evenly and allowed to air dry for 15 min following previously described methodologies [9,39,40]. Control treatments consisted of nylon strips treated with acetone only. Transfluthrin solution was prepared and cloth strips treated at 1200 h in advance of each collection night. Strips were sealed in labeled plastic bags and kept in a light proof box (Soft 6 Cooler, Igloo Products Corp., Katy, Texas) at ambient temperature and humidity in preparation for transport to the field site. In the field, strips were attached to a central wood bean two meters high in the center of designated huts one hour prior to the start of mosquito collections (Figure 3B).

### Mosquito collection

In each experimental hut, mosquitoes were sampled by a two-person team during 12 h overnight collections. Throughout all collection periods, the door of each hut remained closed while the open windows (with interception traps attached) provided the only entry portals for host-seeking mosquitoes. Thirty minutes before dusk (~17.30 h), collection teams entered each structure in order to prepare for trap processing and to establish indoor host cues. Starting at approximately 18.00 h and repeating every 30 min, one collector spent a five minute timed interval aspirating mosquitoes from each window interception trap, collecting for a total of 15 minutes. Trap openings were temporarily blocked with three inch polyurethane foam (Landy’s and Sons, Ltd., Orange Walk Town, Belize) during each collection interval and were immediately re-opened after. Captured mosquitoes were stored in plastic collection cups labeled by time, hut and unique window identifier. Each 12 h collection period was divided into three hour shifts during which collectors took turns alternately processing traps and resting. All anopheline mosquitoes were identified to species using a site appropriate key [41]. Ovarian dissections were conducted on a subset of target vector species collected during the study (a total of 473 *An. vestitipennis* (5% of the total) and 250 *An. albimanus* (9% of the total) to estimate age structure using parity characteristics (parous *vs* nulliparous) [42].

### Follow-on study

After the completion of the push-pull evaluation, an additional four-night follow-on study was performed to test if there was an observable interaction between the spatial repellent and attractant volatiles from the outdoor lure that was diminishing the spatial repellency effect. A two-hut Latin square procedure was employed to evaluate the difference in window intercept catches between two experimental hut conditions: a control hut that utilized the standard push-pull intervention with indoor transfluthrin and outdoor baited CDC LTs and an experimental intervention that utilized indoor transfluthrin and outdoor, non-baited (clean socks) CDC LTs.

### Data analyses

Unless otherwise noted, Excel 2007 (Microsoft, Redmond WA) was used to log_e_ transform the raw numbers of mosquitoes collected and to calculate means, interval endpoints and standard errors. Following standard methods [43], these data were then back-transformed to calculate geometric means of mosquito densities collected, which are presented with standard error of the mean (SEM). To differentiate the mean numbers of mosquitoes entering each of the four huts via window intercept traps, IBM SPSS Statistics v20.0 (Armonk, NY) was used to perform ANOVA with Tukey’s test of Honestly Significant Differences (HSD). An additional post-hoc analysis used Wilcoxon’s Signed Rank test to examine the median nightly differences in mosquito entry between the push and push-pull treatments only. Student’s t-test (α = 0.05) was also used to compare differences in the mean number of mosquitoes trapped in CDC LTs hanging outside huts with and without repellent treatments (pull *vs* push-pull treatments), and to compare the mean numbers of mosquitoes collected in window intercept traps at huts utilizing outdoor CDC LTs with and without lure during the follow-on study.

## Results

Baseline site characterization, performed in July and August of 2012, showed *An. vestitipennis* (72%) and *An. albimanus* (7%) to represent the largest proportions of mosquito species collected (Additional file 2), as had been previously observed in 2011 [29]. Other mosquitoes encountered included *Anopheles crucians* (5%), *Anopheles punctimacula* (1%), *Anopheles gabaldoni* (1%), and a number of other culicines (15%) (Additional file 2) which were not identified to species but included the following genera: *Culex*, *Psorophora*, and *Mansonia*. In addition, pre-intervention collections indicated both hut and collection team comparability for both *An. albimanus* and *An. vestitipennis* (Additional file 3). During treatment evaluations, a total of 21,494 mosquitoes were collected and identified, 15,411 from indoor window interception traps and 6,083 from outdoor CDC LTs (Table 1). Again, the two most abundant mosquitoes collected were *An. vestitipennis* (total n = 9,522) and *An. albimanus* (total n = 2,933). Ambient outdoor temperatures ranged from an average nightly high of 26.9°C (range 21.7°C - 39.8°C) to an average nightly low of 22.3°C (range 16.6°C - 25.1°C) with relative humidity averaging greater than 90% (range 57% - 100%). A spearman’s rank correlation analysis on mosquito densities and climate variables indicated only one significant trend: a positive correlation (Spearman’s rho = 0.525, p = 0.037) between nightly precipitation and the number of *An. albimanus* collected in window interception traps.

**Table 1:** **Nightly mosquito densities collected during the push-pull experimental hut evaluation**

	**Indoor interception trap collections**								**Outdoor baited CDC LT** ^**1**^ **collections**			
	***An. vestitipennis***				***An. albimanus***				***An. vestitipennis***		***An. albimanus***	
**Night**	**Control** ^**2**^	**Pull** ^**3**^	**Push** ^**4**^	**P-P** ^**5**^	**Control**	**Pull**	**Push**	**P-P**	**Pull**	**P-P**	**Pull**	**P-P**
1	417	149	152	223	6	5	3	2	27	47	8	1
2	161	103	47	165	50	2	0	7	23	29	17	5
3	336	95	42	76	106	10	3	5	29	14	34	5
4	160	122	17	22	18	29	6	4	19	6	14	12
5	77	100	45	48	42	27	4	30	3	18	7	35
6	69	68	68	64	329	114	103	171	4	14	22	30
7	43	24	26	81	13	4	11	48	3	19	1	7
8	114	124	63	132	124	123	25	89	9	32	17	26
9	49	37	27	47	29	12	30	40	19	20	13	36
10	189	119	105	127	102	43	40	60	17	54	13	62
11	451	841	397	237	43	20	39	17	232	106	15	15
12	270	396	176	271	9	26	10	13	105	87	25	33
13	168	237	114	214	54	116	44	77	29	39	24	14
14	150	137	15	45	14	18	1	0	30	36	18	5
15	34	53	9	4	1	3	1	1	11	9	3	1
16	25	35	11	30	9	11	12	3	3	6	1	1
Geo Mean (SE)	122 (2.4)	107 (2.5)	48 (2.9)	74 (3.0)	27 (4.1)	18 (3.6)	9 (4.8)	13 (5.7)	16 (3.5)	24 (2.4)	10 (2.9)	10 (3.9)

^1^CDC LT = CDC Light Trap ^2^Control = no treatment.

^3^Pull = outdoor CDC LT alone ^4^Push = indoor spatial repellent alone.

^5^P-P = Push-Pull; combined use of outdoor CDC LT and indoor spatial repellent.

**Figure 4:**
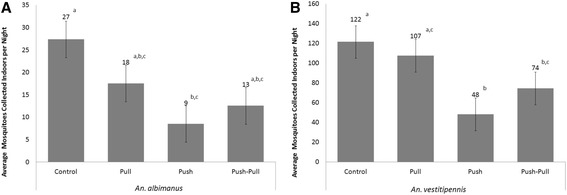
Reductions in mosquito entry. The nightly averages (geometric mean, n = 16) of female mosquitoes collected from window interception traps at each hut for **(A)** *An. albimanus* and **(B)** *An. vestitipennis*. Control = no intervention, Pull = outdoor light trap, Push = indoor spatial repellent, Push-Pull = combined intervention. Error bars show SEM, different lower case letters indicate significantly different means (Tukey’s post-hoc tests of honestly significant differences with α = 0.05).

**Figure 5:**
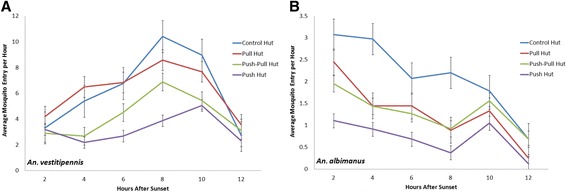
Effect of push-pull components on mosquito hut entry. Aggregate nightly patterns of mosquito entry into control (no treatment), pull (outdoor light traps), push (indoor spatial repellent only) and push-pull (indoor spatial repellent and outdoor baited traps) huts for both **(A)** *Anopheles vestitipennis* and **(B)** *An. albimanus* throughout study (n = 16 nights). Geometric means are presented in 2 h intervals, error bars represent the SEM.

The total number of *An. vestitipennis* and *An. albimanus* collected per night from window interception traps at each of the four experimental huts during the push-pull evaluation is shown in Table 1, with the nightly average number (geometric mean) by treatment shown in Figure 4. For each vector species, the highest mosquito densities (geometric mean per hut) were collected from control huts: 122 (SEM 2.4) per night for *An. vestitipennis* and 27 (4.1) per night for *An. albimanus*. For *An. vestitipennis* the pull treatment (CDC LTs outside windows) did not significantly reduce mosquito entry into huts, 107 (2.5) per night, compared to the control hut, 122 (2.4) per night (Tukey’s HSD p = 0.906) (Figure 4). Similarly, the time of peak entry was unaffected (Figure 5). For *An. albimanus* the effect of CDC LTs on the numbers of mosquitoes entering the hut, though also statistically insignificant (Tukey’s HSD p = 0.488), was greater: a reduction from 27 (4.1) per night in the control to 18 (3.6) per night with the pull intervention (Figure 4). *An. albimanus* hourly entry patterns showed this trend to be consistent throughout the night (Figure 5), as has been previously reported with *An. albimanus* and baited CDC light traps at this field site [29]. The use of the spatial repellent reduced nightly mosquito entry of *An. vestitipennis* by 60% (95% CI: [0.58 – 0.62]) compared to the control hut (48 [2.9] vs. 122 [2.4]; Tukey’s HSD p < 0.001) (Figure 4). Similarly, a reduction of 69% (95% CI: [0.58 – 0.80]) was observed in nightly *An. albimanus* entry, from 27 (4.1) to 9 (4.8) (Tukey’s HSD p = 0.003) (Figure 4). These reductions were significant and consistent throughout the entire collection period (Figure 5).

**Figure 6:**
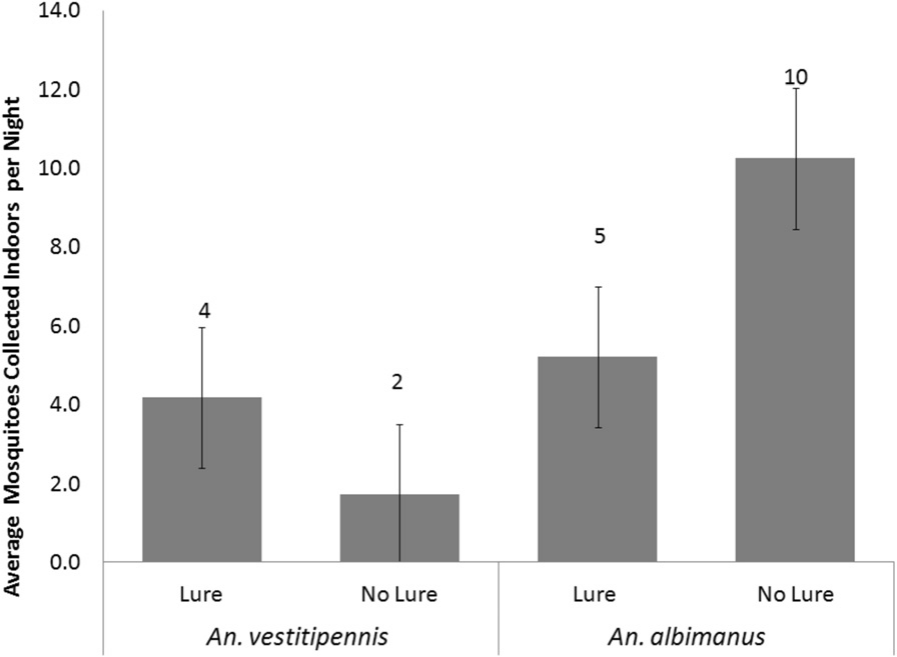
No interaction between outdoor mosquito lure and indoor repellent. The nightly (n = 4) average numbers of mosquitoes collected from indoor window intercept traps in huts deploying baited (Lure) and unbaited (No Lure) CDCLTs. Geometric means are presented, error bars show the SEM. *An. vestitipennis* t-test: t = -0.080, df = 6, p = 0.939; *An. albimanus* t-test: t = -0.866, df = 6, p = 0.420.

The combined push-pull treatment also reduced mosquito entry compared to the control hut, but the impact was slightly less than the effect of using spatial repellent alone (Figure 4). For *An. vestitipennis*, the push-pull reduction in mosquito entry was 39% (95% CI: [0.37 – 0.41]), from an average of 122 (2.4) per night to 74 (3.0) per night (Tukey’s HSD p = 0.047) (Figure 4), while for *An. albimanus* the reduction was 54% (95% CI: [0.30 – 0.68]), from an average of 27 (4.1) to 13 (5.7) per night (Tukey’s HSD p = 0.072) (Figure 4). The reduced repellent effect seen at the push-pull huts, as compared to the push huts, was not statistically significant in terms of the absolute numbers of mosquitoes collected indoors (Tukey’s HSD p = 0.196 for *An. vestitipennis* and p = 0.600 for *An. albimanus*). However, a Wilcoxon signed-rank test comparing the difference of means between the repellent alone and the combined intervention indicated that the trend was significant: the push-pull hut collected more mosquitoes in window interception traps than the push hut on 13 out of 16 night for *An. vestitipennis* (p = 0.016) and on 10 out of 16 nights for *An. albimanus* (p = 0.038). Results from the follow-on study indicate no effect of outdoor CDC LT bait on the spatial repellent effect of indoor transfluthrin: the numbers of mosquitoes captured entering the window interception traps at each hut were not statistically different nor were there any consistent trends observed (Figure 6). For *An. vestitipennis* and *An. albimanus*, no differences in parity rates were observed between mosquitoes captured indoors or outdoors, or in the presence or absence of a spatial repellent, on any night (Additional file 4).

**Figure 7:**
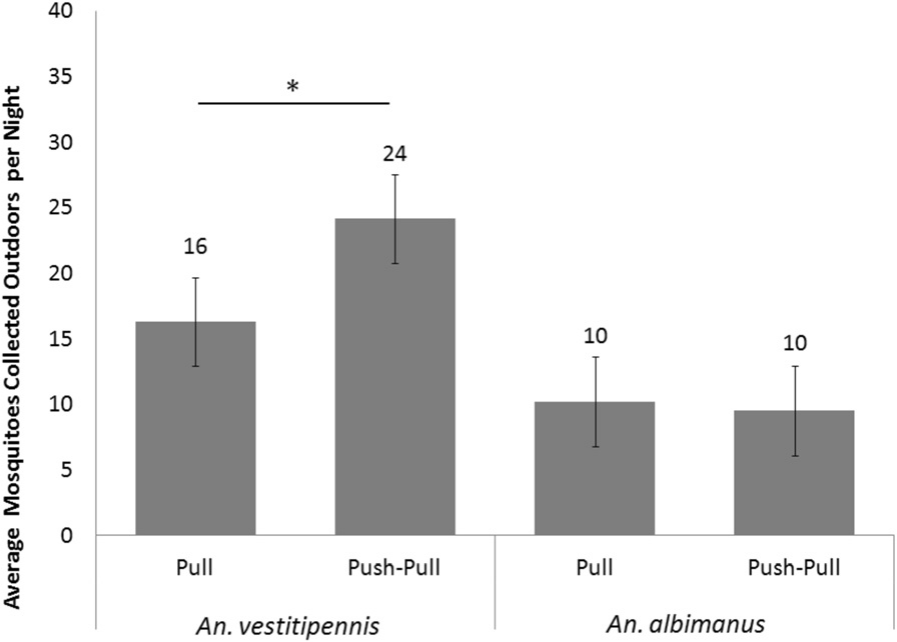
The impact of indoor spatial repellent use on outdoor light trap catches. The nightly (n = 16) average numbers of mosquitoes collected from outdoor CDCLTs at the pull (outdoor light traps only) and push-pull (combined use of indoor transfluthrin and outdoor light traps) huts. Geometric means are presented, error bars show the SEM, * = difference was statistically significant (t-test: t = 1.78, df = 30, p = 0.043).

In the outdoor baited CDC LTs, the indoor spatial repellent treatment increased the nightly density of *An. vestitipennis* captured by 48% (95% CI: [0.22 – 0.74]), from 16.3 (3.5) per night at the pull hut to 24.1 (2.4) per night at the push-pull hut (t = 1.78, df = 30, p = 0.043) (Figure 7); however, no effect was seen in *An. albimanus* populations (10.2 [2.9] vs. 9.5 [3.9] at the pull and push-pull huts, respectively) (Figure 7). Concerned that the lack of effect seen with *An. albimanus* could have been an artifact of the smaller population densities collected, a post-hoc sample size calculation based on observing a response similar in magnitude to that observed with *An. vestitipennis* (an increased CDC LT yield of 48%, or 5 *An. albimanus* mosquitoes per night) using α = 0.05 and β = 0.80 was performed. Calculations indicated a required sample size of 6 nights for both treatments, fewer than the 16 nights evaluated here, supporting the conclusion that this particular differential response between vector species is real and not confounded by the comparatively low numbers of *An. albimanus* collected in CDC LTs.

## Discussion

This pilot study is among the first field-based evaluations of a prototype push-pull system to control naturally occurring vectors of human malaria. The use of multiple experimental huts allowed for the assessment of the impact of each of the components of the intervention, an indoor spatial repellent and an outdoor baited trap, separately and in combination. Additionally, the study site provided an opportunity to assess the impact of the same experimental interventions concurrently on two anopheline species of public health importance, the more endophagic and anthropophagic *An. vestitipennis* and the more exophagic and zoophagic *An. albimanus* [33,44,26,45].

### Outdoor baited light traps: the pull

Data indicate that the use of baited CDC LTs had no statistically significant effect on the entry of either species into the experimental huts, although a moderate decrease in entry for *An. albimanus* was noted. These results are in line with previous observations made at this study site [29] and seem to indicate that an outdoor baited light trap, in the absence of any other mosquito control intervention, was more likely to impact the hut entry behaviors of the more naturally exophagic *An. albimanus*. Conversely, the hut entry behaviors of the endophagic vector *An. vestitipennis*, which is inherently more attracted to the internal environment of an experimental hut, were not impacted by the presence of an outdoor baited light trap.

### Indoor spatial repellent: the push

The impact of indoor transfluthrin emanators was clear and consistent for both species, resulting in sharp reductions in the numbers of host-seeking mosquitoes that entered window interception traps. These results are in line with previous reports of experimental hut studies from Belize in which indoor applications of DDT elicited SR behaviors in *An. vestitipennis* and *An. albimanus* [46,33] and with previous knowledge that transfluthrin repels several other anopheline mosquito species [37,38].

### The combined push-pull system

Against *An. vestitipennis*, results show that the combined push-pull treatment simultaneously reduced mosquito entry into experimental huts, compared to control huts, and increased the numbers of mosquitoes collected in outdoor baited CDC LTs, compared to CDC LTs operating outside huts with no spatial repellent treatment. For *An. albimanus*, results indicate a similar reduction in mosquito hut entry associated with the push-pull treatment, but there was no comparable effect of indoor spatial repellent on increasing outdoor light trap collections.

The use of an indoor spatial repellent significantly and consistently reduced entry into the window interception traps for both target species, whether used alone or in conjunction with outdoor baited light traps. Considering this, along with the statistically negligible (albeit variable) effect on hut entry of outdoor light traps used alone, suggests that the reduced mosquito entry observed at the push-pull huts can be attributed directly to the spatial repellent activity of the transfluthrin treatment. This inference is supported by general observations that, at least given the currently available technologies, spatial repellents can be very effective at reducing local biting pressures [47,48] while trapping of adults has remained largely ineffective at doing so, except when conducted on much larger scales, over longer periods of time and using more sophisticated traps than were evaluated here [49-53]. It should also be noted that the fluctuation in mosquito densities collected over the course of the study, including relatively high numbers collected during the baseline sampling and very low numbers captured during the follow-on study, was expected as the result of normal seasonal variation in mosquito populations in the region and are in line with previous multi-year seasonal studies [27-29]. An abbreviated (6 h) baseline (no intervention) collection performed after the midpoint of the study checked for the possibility of residual repellent effects after hut and trap cleaning, and revealed no decreases in window entry into huts that had previously received repellent treatments compared to control-only huts (Additional file 5).

Interestingly, results in the current study also showed a tendency for the presence of outdoor baited CDC LTs to slightly, but consistently, decrease the repellent effect of indoor transfluthrin against both target vectors. This effect remains largely unexplained, although a follow-on study comparing two huts with spatial repellent treatment and CDC LTs (one hut using baited traps and one hut using unbaited traps) indicated no negative interaction between mosquito lures and the spatial repellent treatment, based on no significant differences in the number of mosquitoes captured in window traps at the two huts. Further study of this tendency is warranted, including whether or not the effect remains if different repellent delivery mechanisms, such as a commercially available product with optimally formulated active ingredient, and/or different trap types and positions are utilized.

There is some contrast between these results and those obtained recently by Menger *et al.* [19] during a somewhat similar push-pull evaluation in Kenya, where a greater impact on mosquito hut entry resulted from baited traps and a lesser effect resulted from the repellent treatment. There are critical differences, however, in the experimental approaches taken that are likely to have influenced study results and preclude direct comparisons. In addition to using a semi-field set-up with the controlled release of laboratory-reared *An. gambiae* into a screened-in area with an experimental hut constructed inside, the study in Kenya utilized different traps (Mosquito Magnet® X [MM-X]), different lures (CO2 in conjunction with a five-component odor blend), and different repellents (the non-pyrethroids para-menthane-3,8-diol, delta-undecalactone, and catnip essential oil) that were deployed outdoors using an active dispersal mechanism [19]. Additionally, the system evaluated here used human collectors inside the huts, while in Kenya an additional baited MMX trap acted as a proxy for an indoor human blood meal [19]. Ultimately, considering these differences, it is exciting that the interpretations of both studies are largely in accord: the combined use of spatial repellents and baited traps in a coordinated push-pull system can achieve targeted control of malaria vectors better than either component used alone. The principle differences in the results reflect mostly variations in experimental design and highlight how the increased complexity of real-world transmission settings can impact intervention results.

One of the novel advantages of a push-pull vector control strategy is the potential to simultaneously decrease human-vector interactions both inside and outdoors. Encouragingly, the densities of *An. vestitipennis* captured in outdoor CDC LTs increased in the presence of the indoor spatial repellent, compared to when CDC LTs were used alone. However, such an effect was not seen with *An. albimanus*. Reasons for this observation might again be explained based on species-specific behaviours. More endophagic species, like *An. vestitipennis*, will be more strongly attracted to the internal environment of an occupied hut during host-seeking. If the indoor environment is found to be unsuitable, it may also be more likely displaced from its endophagic host-seeking path into the immediate peridomestic environment, thus increasing the probability of capture by an outdoor trap positioned adjacent to the host-occupied structure. Upon detection of an unsuitable indoor environment, an exophagic species like *An. albimanus* may simply continue to search for a blood meal in a wider area outdoors, not impacting (or perhaps lowering) the probability of contact with the same outdoor traps.

Finally, though only a subset of *An. vestitipennis* and *An. albimanus* were age graded via ovarian dissection during each collection, it is important to mention that there were no obvious differences in the crude age structures of target vector populations with regards to the location of their capture on any night. While parity rates did fluctuate temporally throughout the duration of the study, there was no evidence that any of the interventions had a differential impact on nulliparous or parous mosquitoes.

### Field optimization

The goal of this study was to evaluate general proof-of-principle for a combined push-pull strategy for the control of natural malaria vectors in the field. As such, the best locally available tools (repellent and trap type) were selected for use in a multi-component intervention at an experimental hut site in northern Belize, Central America. While the results are encouraging, further optimization and validation of the approach are clearly warranted. It is difficult to imagine a mature public health intervention that utilizes repellent treated nylon strips and baited CDC LT’s in the current prototype configuration, but discussion of the present data does highlight that combined push-pull effects are possible thereby providing a critical basis for continued study and development of the strategy. Based on this prototype, the clearest indication for use of an outdoor trap in addition to an indoor repellent might be when there is documented transmission outdoors in the peridomestic area around homes. It is also likely, however, that any capture of mosquitoes would impact overall vector densities by removing a proportion of the population through a mechanical (non-insecticidal) mechanism that would not select for insecticide resistance. Current modeling efforts are beginning to tackle many of these questions and they are active points of discussion in the community, but more work is clearly needed and a thorough discussion of them is well beyond the scope of this study.

Indeed, the true field optimization of a push-pull system for vector control will involve a host of complex issues, including under which circumstances the use of both the repellent and trapping components would be preferable to the use of repellent alone. This will require an understanding of local transmission dynamics, such as the differentiation between indoor and outdoor biting rates and risks of infection, as well as cost benefit analyses, assessments of ease and general feasibility studies. The validation of the strategy in any location will have to include rational selection of the best available tools in relation to that particular environment, and a thorough understanding of the local vector (and human) ecologies will be essential in shaping each intervention, e.g. which repellent products and traps to use and where to position them relative to the population at risk and vector breeding, resting and feeding sites. Considering this, it is doubtful that an optimized push-pull intervention targeting an endophagic species like *An. vestitipennis* will be exactly the same as an intervention tailored to target a more exophagic species like *An. albimanus*. Also, it is likely that in order to truly achieve maximum impact from a push-pull strategy we need to develop better traps and new repellents. Again, investigations of these complex issues are ongoing and continued discussions will be critical to the further development of novel vector control strategies including push-pull.

## Conclusion

The experiments reported here demonstrate the potential for push-pull strategies to reduce the probability of human-vector interactions both inside (by reducing mosquito entry) and outside (by increasing the yields of outdoor baited traps) of homes, and support further investment into the optimization and validation of the approach for disease vector control. However, the variation in effect seen on different target species highlights the need to identify the underlying behavioral ecology of local vectors to tailor the strategy to different transmission settings. Additionally, further elucidation of the species-specific mechanisms that drive mosquito responses to spatial repellent chemicals and baited traps is needed to properly evaluate the potential role for push-pull vector control strategies as part of any malaria prevention programme.

## Additional files

Additional file 1:
**The Graeco-Latin square study design.** Each specific combination of experimental treatment and collection team was rotated through each one of the huts. Additional file 2:
**Adult female mosquito composition at the study site.** From baseline (pre-intervention) characterization of the site from July to August, 2012. Additional file 3:
**Baseline (pre-intervention) comparability of experimental huts.** During the baseline characterization of mosquito activity, no significant differences were observed in the number of mosquitoes collected according to (A) hut location (ANOVA: F = 0.330, df = 15, p = 0.804 for *An. vestitipennis*; F = 0.484, df = 15, p = .699 for *An. albimanus*) or (B) collection team (ANOVA: F = 0.333, df = 15, p = 0.802 for *An. vestitipennis*; F = 0.210, df = 15, p = .887 for *An. albimanus*). Nightly (n = 4) geometric means are shown; error bars represent the standard error of the mean. Additional file 4:
**Parity rates for subset of captured** ***An. vestitipennis*** **and** ***An. albimanus*** **that were age graded via ovarian dissection, by collection night.**
Additional file 5:
**Midpoint (06 October 2012) baseline collections.** Window trap densities were not diminished in huts that had previously received repellent treatments, indicating no residual effects after cleaning.
