# Supplementary figures and images for: The field evaluation of a push-pull system to control malaria vectors in Northern Belize, Central America
Source: Malar J. 2015 Apr 29;14:184. doi: 10.1186/s12936-015-0692-5 (PMC4425932; doi:10.1186/s12936-015-0692-5)

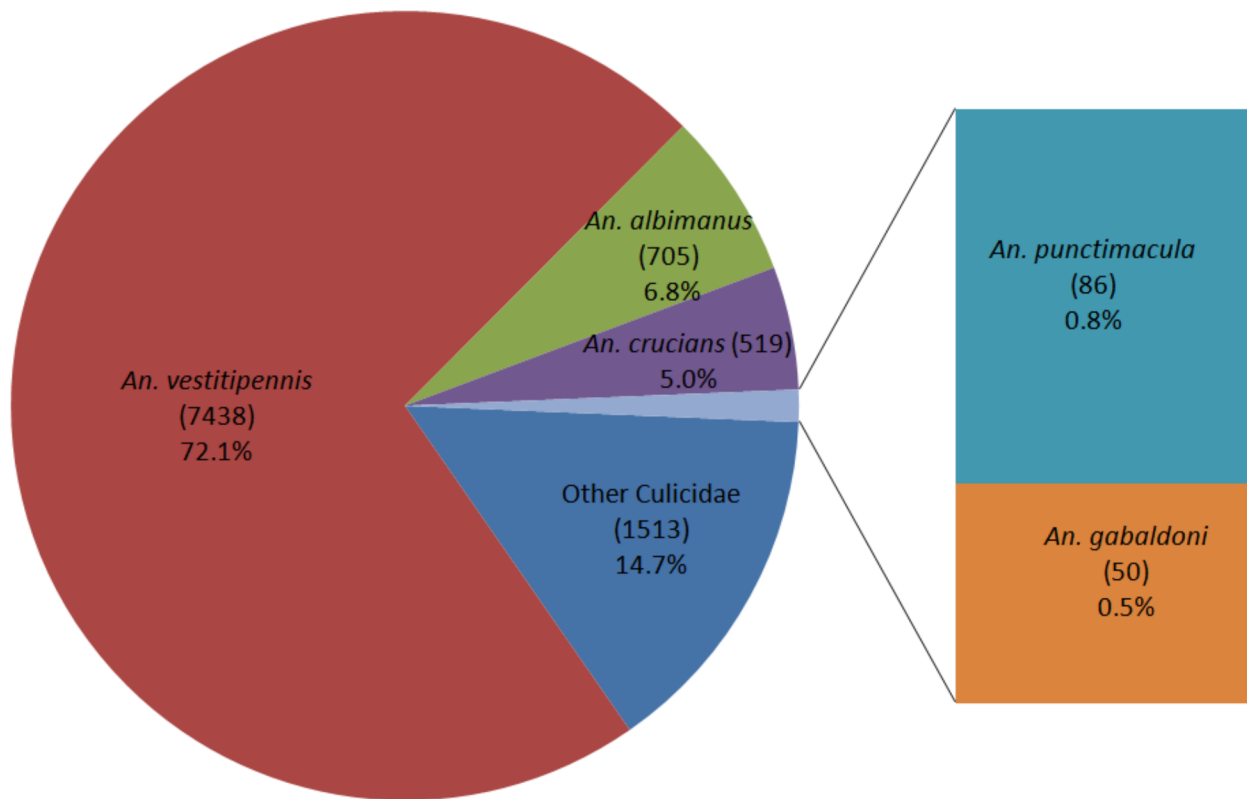

Total Collected = 10,311

Supplement: Additional file 2: — Adult female mosquito composition at the study site. From baseline (pre-intervention) characterization of the site from July to August, 2012. [file 12936_2015_692_MOESM2_ESM.pdf]
