# Supplementary material for: The field evaluation of a push-pull system to control malaria vectors in Northern Belize, Central America
Source: Malar J. 2015 Apr 29;14:184. doi: 10.1186/s12936-015-0692-5 (PMC4425932; doi:10.1186/s12936-015-0692-5)
Supplement: Additional file 3: — Baseline (pre-intervention) comparability of experimental huts. During the baseline characterization of mosquito activity, no significant differences were observed in the number of mosquitoes collected according to (A) hut location (ANOVA: F = 0.330, df = 15, p = 0.804 for An. vestitipennis; F = 0.484, df = 15, p = .699 for An. albimanus) or (B) collection team (ANOVA: F = 0.333, df = 15, p = 0.802 for An. vestitipennis; F = 0.210, df = 15, p = .887 for An. albimanus). Nightly (n = 4) geometric means are shown; error bars represent the standard error of the mean. [file 12936_2015_692_MOESM3_ESM.pdf]

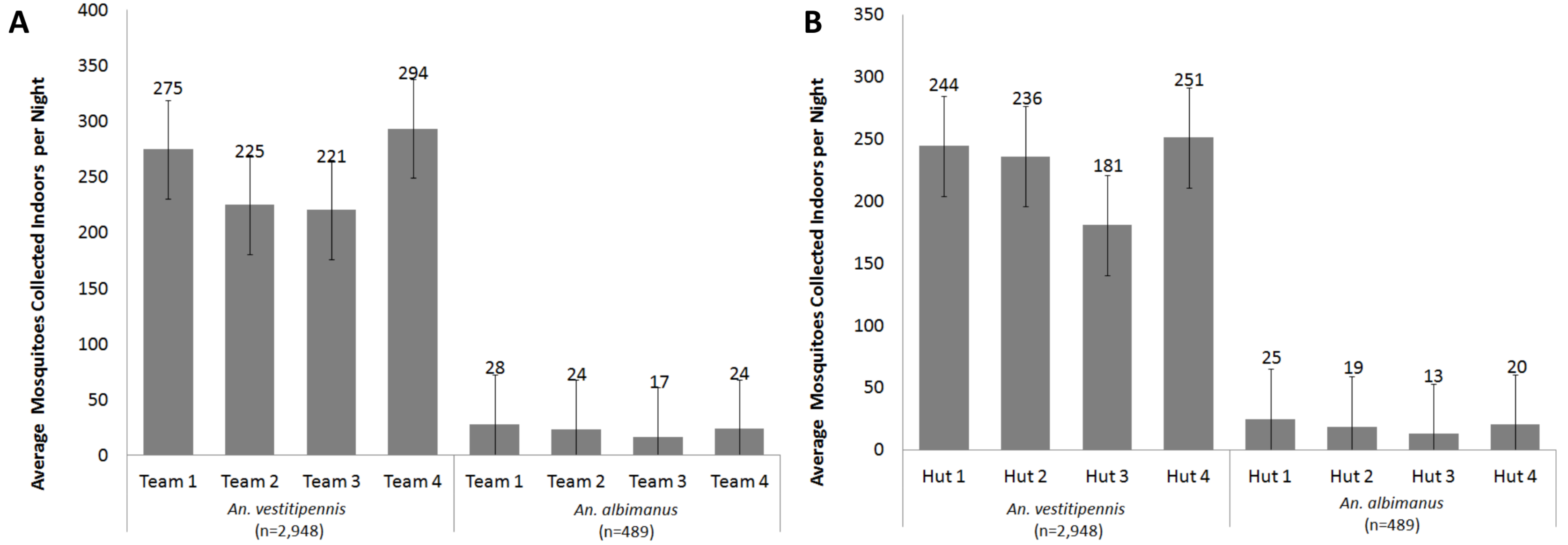

A
B
Average Mosquitoes Collected Indoors per Night
400
350
300
250
200
150
100
50
0
275
225
221
294
28
24
17
24
Team 1
Team 2
Team 3
Team 4
Team 1
Team 2
Team 3
Team 4
An. vestitipennis
(n=2,948)
An. albimanus
(n=489)
244
236
181
251
25
19
13
20
Hut 1
Hut 2
Hut 3
Hut 4
Hut 1
Hut 2
Hut 3
Hut 4
An. vestitipennis
(n=2,948)
An. albimanus
(n=489)
